# Dynamic Responses of a Single-Axle Trailer When Driving Over a Road Obstacle

**DOI:** 10.3390/s25175246

**Published:** 2025-08-23

**Authors:** Dalibor Barta, Miroslav Blatnický, Alyona Lovska, Sławomir Kowalski, Aleš Slíva, Ján Dižo

**Affiliations:** 1 Department of Transport and Handling Machines, Faculty of Mechanical Engineering, University of Žilina, Univerzitná 8215/1, 010 26 Žilina, Slovakia; dalibor.barta@fstroj.uniza.sk (D.B.); miroslav.blatnicky@fstroj.uniza.sk (M.B.); alyona.lovska@fstroj.uniza.sk (A.L.); 2 Faculty of Engineering Sciences, State University of Applied Sciences in Nowy Sącz, 1a Zamenhofa Street, 33-300 Nowy Sącz, Poland; skowalski@ans-ns.edu.pl; 3 Institute of Transport, Faculty of Mechanical Engineering, VSB-Technical University of Ostrava, 17 Listopadu 15/2172, 70800 Ostrava, Czech Republic; ales.sliva@vsb.cz

**Keywords:** vehicle–trailer combination, single-axle trailer, safety, individual obstacle, acceleration, experiments

## Abstract

Trailers for passenger cars are often used for the transportation of goods. There are various trailer designs. Most trailers are equipped with axles, which include swinging arms and are suspended by rubber segments. Observations have revealed that empty trailers have unfavorable driving properties when they are driven on uneven roads, for example, the wheels could jump off the road. Such a situation is dangerous because it is not possible to transmit any contact forces (longitudinal, lateral, or vertical) between the wheel and the road. The goal of the present research was to measure acceleration generated in a single-axle trailer when driving over a road obstacle. Measurements were conducted in a non-public area to avoid the risk of accidents. Acceleration was recorded using two accelerometers placed on the single-axle trailer frame above the wheels’ axle of rotation. Tests were performed using a vehicle–trailer combination at the chosen driving speeds, and the results for driving speeds of 20 and 30 km/h are presented. Wood plates with a height of 25 and 50 mm were used as an artificial road obstacle. The single-axle trailer was loaded with gravel bags weighing 0 to 300 kg. The measurements revealed that heavier trailer loads and lower driving speeds are safer for trailer operation. Furthermore, the measurements also demonstrated that the wheels were significantly more likely to jump off the road with a 0 kg load and low driving speed.

## 1. Introduction

Today, trailers are often used with road vehicles. There are many types of trailers that are usually chosen used based on the cargo being transported. Trailers can be used to transport various types of cargo, such as construction materials, loose bulk materials, motorized and non-motorized vehicles, and even animals.

Trailers are usually manufactured in a manner similar to cars, i.e., using welding technologies, but their production processes are simpler [1,2,3]. Steel is the most common material used to produce the main components of the chassis to ensure the long-term, reliable, and safe operation of the trailers. The chassis and its components, the suspension, axle, wheels, and tires, are the most important parts of a trailer [4,5]. Their function is to ensure contact between the trailer and the road surface and to ensure proper driving characteristics. The individual components of a trailer chassis should be designed to withstand various loads, which are mainly due to road irregularities. They need to be checked regularly; failure to do so may lead to negative impacts on the driving characteristics.

There are two main criteria for the assessment of vehicles: passenger comfort and driving safety. Driving safety is investigated in terms of stability, maneuverability [6], and tire/road contact forces. Driving comfort and force ratios are affected by vehicle oscillations and vibrations. A vehicle–trailer combination represents a complex mechanical system and problems with vehicle and trailer responses during operation are relatively difficult to solve. Therefore, researchers have focused on studying individual problems; a review of selected sources is presented below.

Comfort is largely related to the towing vehicle, which also transports passengers. As described in [7], the human body is sensitive to transmitted accelerations. Accelerations can be measured using a suitable method [8]. Driving comfort is investigated through experimental or simulation tests, depending on requirements and available resources [9,10,11].

Acceleration is a consequence of a vehicle and a trailer being driven on a road with irregularities. These road irregularities cause excitation forces, which lead to vehicle and trailer movement. These movements are undesirable, and efforts are made to minimize them [12,13,14]. The subject of this research is a vehicle–trailer being driven on a road with a single irregularity, which is represented by a road bump. As noted in [15], road potholes can pose a danger to traffic safety, cause damage to vehicles, and increase the accident risk, especially under low-visibility conditions. Therefore, a methods have been proposed to study and reduce their impacts. According to [16], new drivers decelerate to overcome bumps at lower speeds. Additionally, another study [17] showed that speed bumps on roads negatively affect the braking distance. Acceleration generated while driving on road irregularities or speed bumps can be measured using accelerometer sensors placed on the car or trailer depending on the purpose of the experiment. There are also specialized measuring devices that can detect and process of acceleration signals. This data can be used to assess road quality and can be input into a simulation model [18,19,20,21].

It is necessary to minimize the vibrations and oscillations of the vehicle as well as the trailer. The suspension systems of the vehicle and trailer are closely connected in relation to vibrations and oscillations. The damping of the mechanical systems of the vehicle and trailer is another crucial factor that influences their driving properties [22]. The different types of suspension systems are described below. It is worth mentioning that there is interesting research on the possibility of harvesting a energy from a suspension system. Previous studies [23,24] did not focused on harvesting energy from trailer suspension systems but this idea could be developed.

The presence of a trailer also affects the aerodynamics of the entire vehicle–trailer combination. While the vehicle itself is currently designed to minimize aerodynamic drag [25,26,27], when a trailer is attached, the aerodynamic properties of the entire combination are significantly reduced. Therefore, trailer manufacturers are trying to minimize aerodynamic drag through the optimal design of trailers.

A significant factor affecting driving characteristics is the load of the trailer, as well as the placement and attachment of the transported object on the trailer’s loading surface [28,29]. The load weight has the greatest impact on the center of gravity of both the trailer and the towing vehicle. The maximal load capacity of a trailer should not be exceeded under any circumstances.

Human factors, e.g., the driving style, also affect driving characteristics. The speed of the vehicle–trailer combination should be adapted to these factors and to the surrounding conditions, such as the condition of the road surface and the weather.

The present research measured the dynamic load of a single-axle trailer, i.e., the value of the load when driving over an artificially created road obstacle consisting of a wood plate on a road. The given measurements were carried out on a flatbed trailer. The main task was to determine the loads and driving speeds that produce higher acceleration values [30].

## 2. Trailer Towing

Before a trailer can be used on the road, it is necessary to consider the parameters of the towing vehicle and selected trailer from a safety as well as comfort point of view [31]. The key parameters of a towing vehicle are the engine power and braking force. The maximal weight and maximal load capacity are the key factors for a trailer. The type of trailer should be chosen considering the maximal capacity of the towing vehicle.

The power required to tow a trailer depends on several factors, and especially on the total driving resistance of the trailer. Driving resistance refers to a combination of air drag [32], acceleration resistance, rolling resistance, and, in certain cases, climbing resistance. The values of the individual resistances depend primarily on the total weight and type of trailer. Other factors affecting driving resistance include the condition of the road, the speed, and the resistance of frontal area of the trailer.

Brakes are an essential safety component for transport vehicles [33]. When a vehicle–trailer combination includes an unbraked trailer, only brakes of the towing vehicle are responsible for braking the entire vehicle–trailer combination. The towing vehicle must be able to stop without problems within the prescribed braking distance—even if its brakes are being subjected to great demands and overheating or damage—or an accident may occur [34,35]. In the case where a braked trailer is included in a vehicle–trailer combination, the brakes of the trailer are also in operation during braking [36]. These brakes significantly contribute to reducing the braking distance. Nowadays, a widely used type of trailer brake system is overrun brakes. The overrun brake design is based on the fact that when the vehicle combination slows down, the trailer has a higher speed than the towing vehicle due to the effect of inertia, which causes the towing vehicle to push forward. The single-axle trailer used for the research experiments in this study was equipped with an overrun brake.

## 3. Factors Affecting the Driving Properties of a Single-Axle Trailer

The structure of a trailer consists of several parts that work together during driving and their main task is to ensure proper operation and driving properties. These parts include a trailer frame, an axle, wheels with tires, a suspension system, a drawbar, and a superstructure. The trailer frame is the main load-bearing part and connects all the components. It transmits the load of the trailer, the vibrations due to driving on road irregularities, and all the reactions due to acceleration and deceleration or other additional loads. It should be rigid but sufficiently flexible at the same time to withstand torsional and bending loads [37]. The frame should be lightweight to decrease the load capacity. The trailer axle is another load-bearing structural part, which connects two opposite wheels. It transmits the curb weight together with the payload. Furthermore, it transmits the forces generated during driving including centrifugal forces and braking forces in the case of a braked axle. It should be designed to have a long lifespan [38]. The wheels and tires are the contact points between a trailer and the road. Their main task is to transmit the load of the trailer to the road and to keep the trailer moving in the desired direction. Tires also dampen vibrations during driving and they transmit braking forces in the case of a braked axle. A suspension system is very important structural part of a trailer. It absorbs and dampens impacts and shocks caused by driving on uneven road surfaces. This protects the chassis parts from damage from vibrations [39]. A suspension system includes flexible components, which allow for the movement of the individual components of an axle. A drawbar is a crucial link between a towing vehicle and a trailer. It transfers towing forces and ensures stability during driving. A superstructure is placed on top of the frame and it is designed to enclose goods. Its main function is to facilitate the safe and efficient transport of specific types of goods.

The driving properties of a trailer are influenced by several factors. Some of these factors cannot be easily changed, which mainly includes the road conditions, e.g., roadway irregularities, such as potholes and beaten tracks, and the surface quality [40,41]. However, there are also factors that can be changed, such as the trailer design and structure (suspension system [42], wheels, tires, etc.). The load of a trailer and position of the load in the trailer loading area are other important factors that greatly affect its driving properties. Last but not least, human factors also affect the driving properties of a vehicle–trailer combination.

### 3.1. Suspension System

There are various types of suspension systems used in trailers [41]. One type consists of leaf springs, which is mainly used in trailers designed to transport heavy loads. The disadvantage of leaf springs is their relatively complex design and higher demands for both maintenance and repair. The biggest advantage of leaf springs is their self-damping effect. This means that in practice, additional shock absorbers are often not needed [43].

Coil springs are a relatively affordable type of suspension component. Unlike leaf springs, a coil spring does not have a self-damping effect and requires additional shock absorbers. A coil spring is relatively simple in terms of design and requires almost no maintenance. However, coil springs are not widely applied in trailer chassis because their size takes up a significant amount of space.

**Figure 1:**
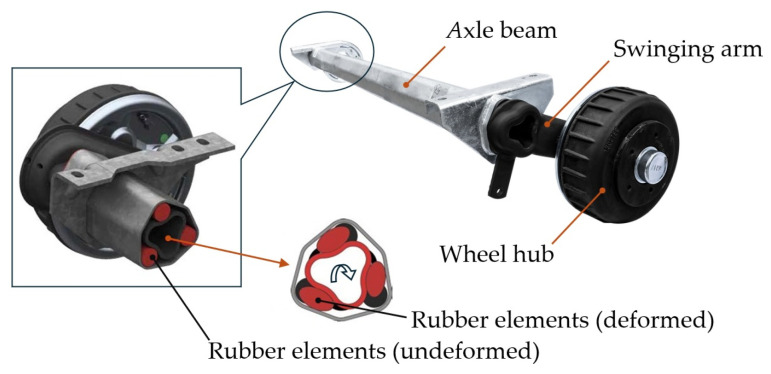
Illustration of trailer axle including torsion bars [44,45].

The most widely used trailer suspension system uses torsion bars. They are usually placed in bundles of two to three bars either in the transverse or longitudinal direction and are mainly subjected to torsion (Figure 1). They are two versions, with a circular or square cross-section. They consist of a swinging arm that is rotationally connected to the axle beam. Rubber elements are inserted between them, as shown in Figure 1. These rubber elements ensure a flexible connection between the swinging arms and the axle beam and a certain level of damping.

From a structure point of view, this is a simple type of suspension that requires almost no maintenance. Another significant advantage of this type of a suspension system is that it requires little space and the individual components (mainly rubber elements) are protected against the weather conditions. When a higher level of driving properties is required, an additional shock absorber is also included, similar to coil springs. The tested single-axle trailer in this study was equipped with this type of suspension system.

### 3.2. Tires and Tire Pressure

Wheels, tires, and tire pressure are closely related to the driving properties of a trailer. Their main function is as the point of contact between the vehicle and road [46,47]. They also function in suspension.

Recently, standard freight single-axle trailers are equipped with wheels consisting of a steel rim and a tubeless tire, with a diameter of 10′ to 14′. Wheels with large diameters are exclusively used for this type of trailer and have lower rolling resistance. The cargo is usually loaded between these wheels to reach a lower loading edge.

**Figure 2:**
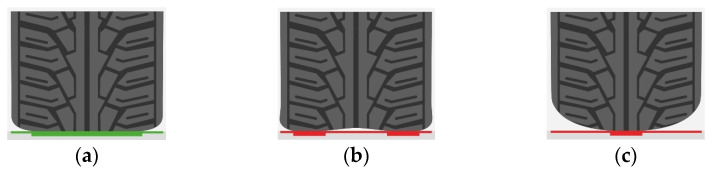
Scheme showing degrees of tire inflation [48]: (**a**) correct inflation; (**b**) underinflation; (**c**) overinflation.

An important factor in terms of both safety and maintenance of the ideal driving characteristics is the tread and inflation of the tires (Figure 2), which should be checked regularly during use [48].

Improper tire conditions, e.g., worn treads and incorrect inflation, can lead to extreme deterioration of the driving properties and in the worst case, it can even lead to fatal consequences.

The single-axle trailer used in this study was equipped with wheels with a steel rim and tubeless tire with a diameter of 14′ and a width of 185 mm. The tests were performed using a tire pressure of 1.6 to 2.8 bar, as recommended by the manufacturer.

### 3.3. Trailer Loading

Another important factor that significantly affects the driving properties of a vehicle–trailer combination is the placement of the transported cargo in the loading area. The total weight of the vehicle combination is particularly important and the maximum permissible weight of both the towing vehicle and the trailer cannot be exceeded. Likewise, the maximal permissible weight per axle of the towing vehicle cannot be exceeded.

During transport, heavy cargo must be placed and properly secured in the loading area of the trailer [49]. The proper placement of the cargo can be verified by a simple check. When the cargo is properly distributed, it is possible to lift the drawbar of the loaded trailer without any problems and without external assistance. However, when the cargo is not placed and secured properly, it may move during transport, which can lead to negative effects.

When the weight of the load is concentrated behind the axle of a trailer, it acts on the trailer drawbar, resulting in negative vibrations in the vehicle–trailer combination [50,51]. Incorrect loading or placement of the load can also lead to instability [52,53,54] of the vehicle–trailer combination during braking of the front axle of the vehicle, as mentioned in the previous section.

When loading a trailer, it is also necessary to pay attention to the vertical load of the tow bar. This should not be exceeded under any circumstances for safety reasons and to maintain the ideal driving properties of the vehicle–trailer combination. The maximal permissible load on the tow bar of the towing vehicle should be stated in the vehicle passport.

## 4. Main Research Goals

The main goal of this research was to measure the acceleration of a single-axle trailer using accelerometers placed over the left and right wheels. The single-axle trailer was towed by a passenger car. The measurements were performed in a closed area without public access. The trailer was loaded with different gravel loads for the tests, and the vehicle–trailer combination was driven at a low driving speed. The acceleration of the towing bar of the vehicle was also measured. The main purpose of the study was to evaluate how acceleration was generated while driving over a single road obstacle under different driving conditions. The road obstacle used is similar to road bumps that are encountered in the real world.

Only the research results for selected driving conditions are presented in the subsequent sections:

- Measurements using a 25 mm high obstacle, a driving speed of 30 km/h, and a load of 0 or 300 kg (Section 6.1) with underinflated tires (Section 6.1.1);
- Measurements using a 25 mm high obstacle, a driving speed of 30 km/h, and different trailer loads (Section 6.2) or a load of 300 kg (measuring acceleration in the *x*-axis direction) (Section 6.2.1);
- Section 6.3 describes the measurements using a 50 mm high obstacle and a speed of 20 km/h.

## 5. Measurement of the Dynamic Load on a Trailer on an Uneven Road Surface

**Figure 3:**
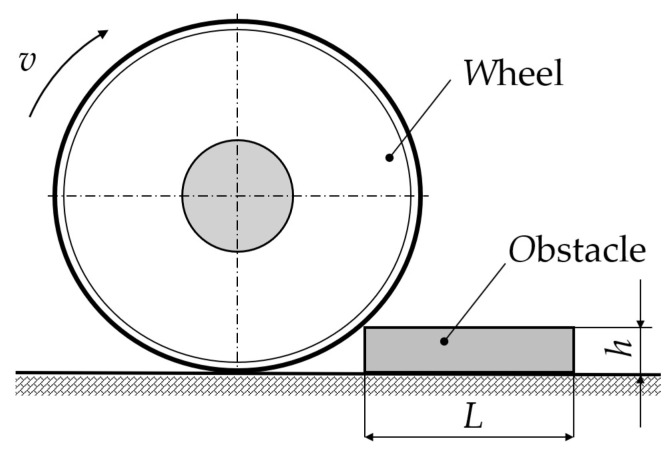
Scheme showing trailer wheel driving over road obstacle.

The goal of study was to determine which acceleration acts on the loaded trailer and its chassis components and how they affect the driving properties when driving over a road obstacle. The measurements were performed using a Corrsys Datron DAS-3 device and the corresponding sensors that are designed to measure acceleration. The tests were carried out in a parking lot on the grounds of the university. The vehicle–trailer combination consisting of a towing vehicle and a single-axle trailer. The obstacle consisted of two wooden boards with a length of 2000 mm, height *h* of 25 mm, and width *L* of 100 mm (Figure 3). Thus, 25 mm and 50 mm high obstacles were used in the experimental tests. The load of the trailer was simulated using bags filled with gravel, with each bag containing a load of 25 kg. The maximum load weight used was 300 kg (12 bags) of gravel that were evenly distributed in the loading area of the single-axle trailer.

### 5.1. Experimental Tests

The aim of the tests was to characterize the influence of the trailer load on the trailer’s behavior when driving over a road obstacle. For this purpose, several series of measurements were performed, while varying the factors that influence driving properties: driving speed (Table 1 and Table 2), trailer load (Table 3 and Table 4), and inflation degree of the trailer tires. Selected research results are discussed in Section 6.

### 5.2. Vehicle–Trailer Combination

**Figure 4:**
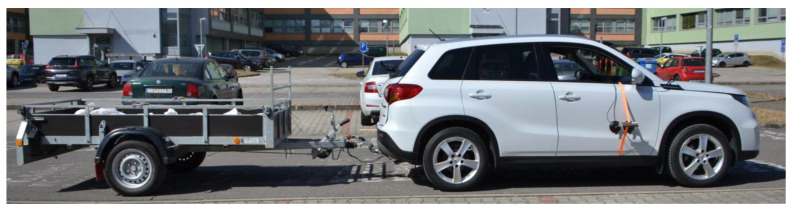
Vehicle–trailer combination used in experimental tests.

The vehicle–trailer combination (Figure 4) consisted of a M1 category towing vehicle and an O2 category single-axle trailer. The towing vehicle is a passenger motor vehicle; its parameters are listed in Table 5. The single-axle trailer is a flatbed trailer; its parameters are presented in Table 6.

### 5.3. Measuring Instruments

The Corrsys Datron DAS-3 (Corrsys-Datron Sensorsysteme GmbH, Wetzlar, Germany; currently: Kistler Instrumente AG, Winterthur, Switzerland) measuring device consists of several parts, which enable the measurement and recording of parameters such as speed, acceleration, traveled distance, measurement time, pressure, force, etc. The device consists of sensors, a display unit, a measuring center, a power supply, and various cables.

The measured values are recorded in volts, which the device then converts into a format that enables the analysis of the results. The data were recorded on a memory card marked CF. The measured data were converted into text files using a computer. The recording frequency was set at 100 Hz during the measurements.

The following data were recorded during the experimental tests: driving speed, distance travelled, time, and acceleration of the single-axle trailer.

#### 5.3.1. Measuring Station

**Figure 5:**
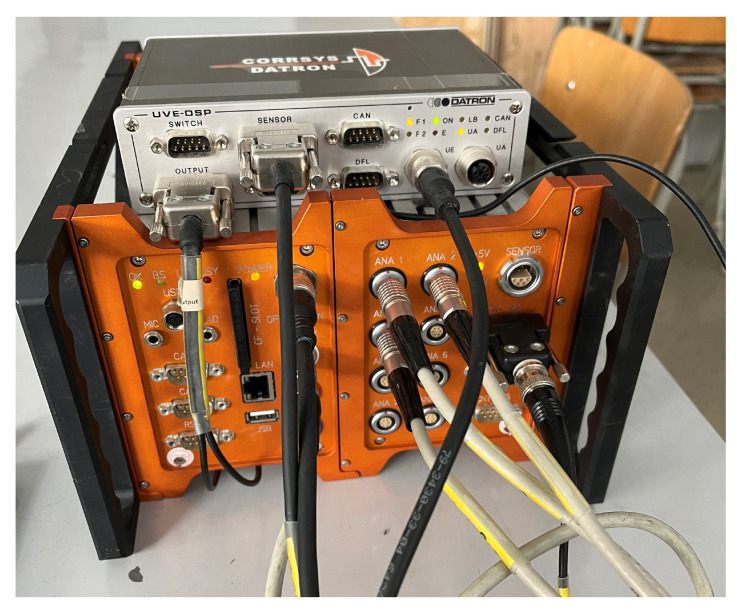
Photo of a measuring station.

The measuring unit (Figure 5) was used to record the measured data to a CF memory card. The measuring unit receives a significant number of different inputs:

- Two digital inputs;
- Two independent CAN inputs;
- Six counter inputs;
- Eight analog inputs.

It is important to correctly set the conditions for the start and end of the measurements. Since the device can measure several quantities simultaneously, the above conditions may influence the measurement accuracy.

The start and end of the measurements can be initiated using the following methods:

- Manually, using the START and STOP buttons located on the control display;
- By switching the sensor to the sensor that is affected by the stimulus;
- By changing the quantity;
- Based on a set time—the measurement starts and terminates after the set time limit.

**Figure 6:**
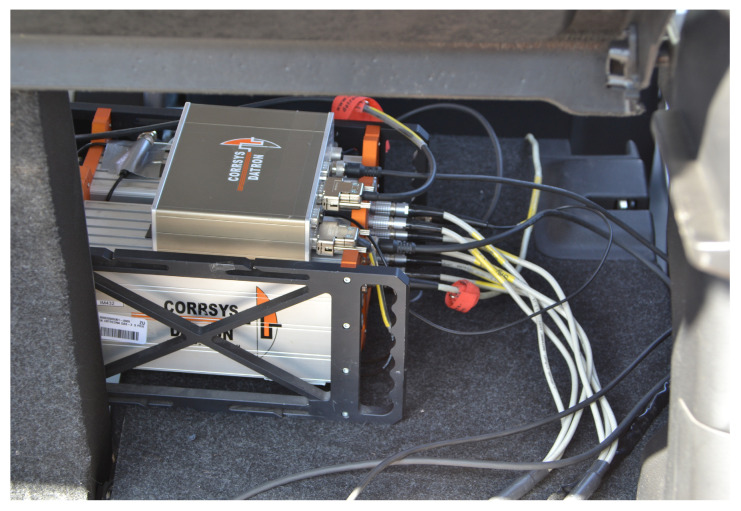
Measuring station in vehicle (in luggage space).

The measuring station was located in the vehicle (Figure 6) and its power was supplied through the vehicle’s on-board network by connecting it using the terminals to the battery contacts of the towing vehicle.

#### 5.3.2. Microstar Non-Contact 1-Axis Microwave Sensor

**Figure 7:**
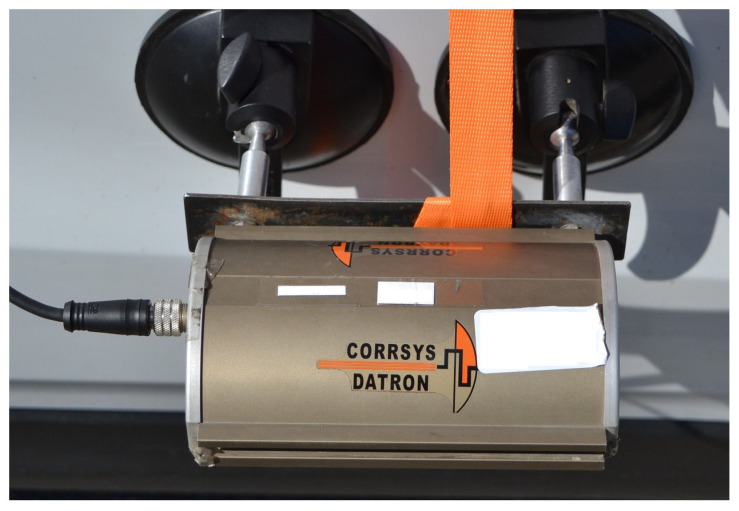
Microstar Non-Contact 1-Axis Microwave sensor.

The Microstar Non-Contact 1-Axis Microwave sensor is a contactless sensor (Figure 7), which was connected to the measuring center. It records data on the distance covered and the speed of the vehicle. The sensor functions based on the Doppler effect.

The basic parameters of the sensor are as follows:

- Range: 0.5 to 400 km/h;
- Height above road surface: 300 to 1200 mm;
- Power supply power: 9 to 32 V DC, 10 W.

**Figure 8:**
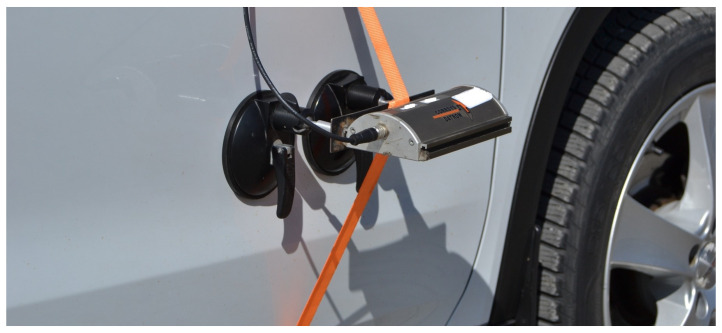
Sensor attached to vehicle.

The sensor was attached to the right door of the vehicle (Figure 8), using suction cups and secured with a tension strap, approximately 700 mm above the road surface during the tests.

#### 5.3.3. TAA-3206M4 Sensor

**Figure 9:**
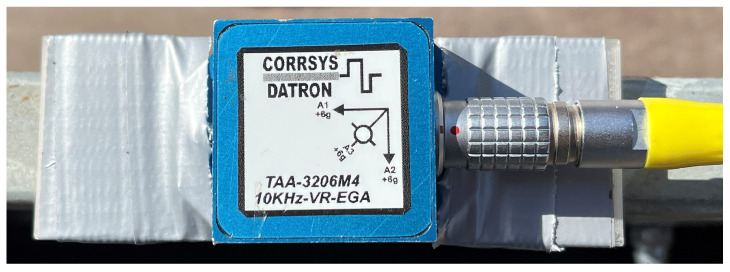
TAA-3206M4 sensor.

**Figure 10:**
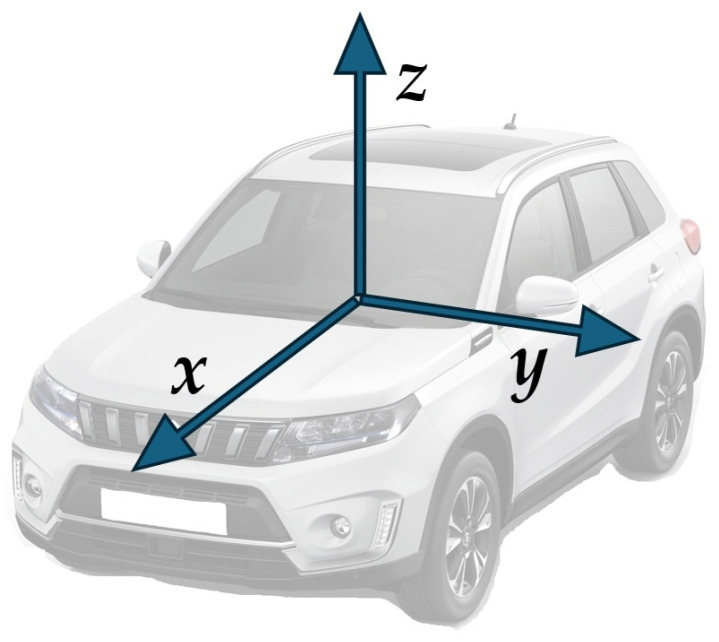
Coordinate system.

The TAA-3206M4 sensor is an accelerometer (Figure 9), which can measure and capture the acceleration in three axes, i.e., *x*, *y*, and *z*, and the gravitational acceleration in the direction of the *z*-axis, i.e., in the vertical direction (Figure 10).

The orientation of the axes was as follows (Figure 10):

- *x*-axis (A1)—used to measure horizontal movement in the direction or opposite direction of vehicle movement;
- *y*-axis (A2)— used to measure horizontal movement perpendicular to the direction of vehicle movement;
- *z*-axis (A3)— used to measure vertical movement.

**Figure 11:**
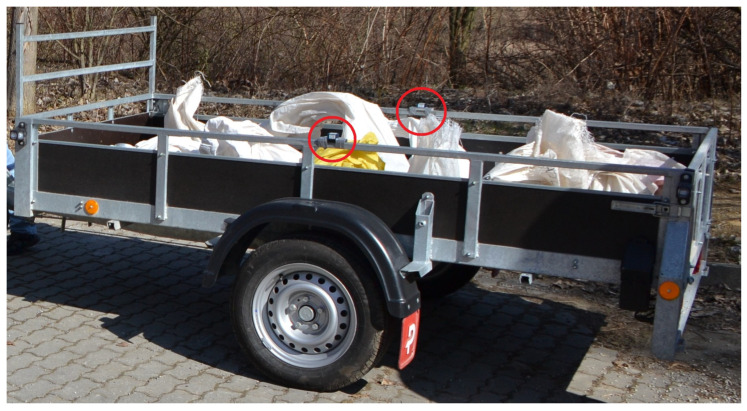
Single-axle trailer with mounted sensors.

Two sensors mounted on the trailer directly above the wheels, as shown in Figure 11, were used to perform the measurements.

#### 5.3.4. Corrsys Datron Pedal Force Sensor

**Figure 12:**
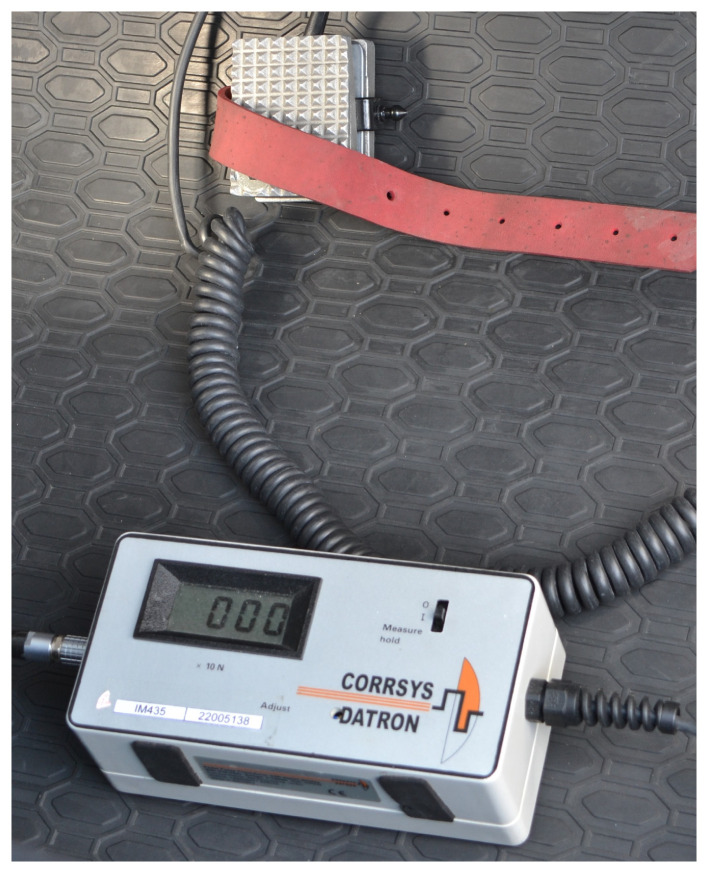
Corrsys Datron Pedal Force sensor.

The Corrsys Datron Pedal Force sensor is a strain gauge sensor (Figure 12). It is able to record the force exerted on the pedal when it is stepped on. It can be attached to any pedal in a vehicle. For the purposes of this research, it was placed under the passenger’s feet and served as a signaling unit for recording the moment the measurement started. The value of the measured force is given in Newtons [N].

The basic parameters of the force sensor are as follows:

- Range: 0–1500 N;
- Measurement accuracy: 3% (average); 7% (minimum).

#### 5.3.5. Control Display

**Figure 13:**
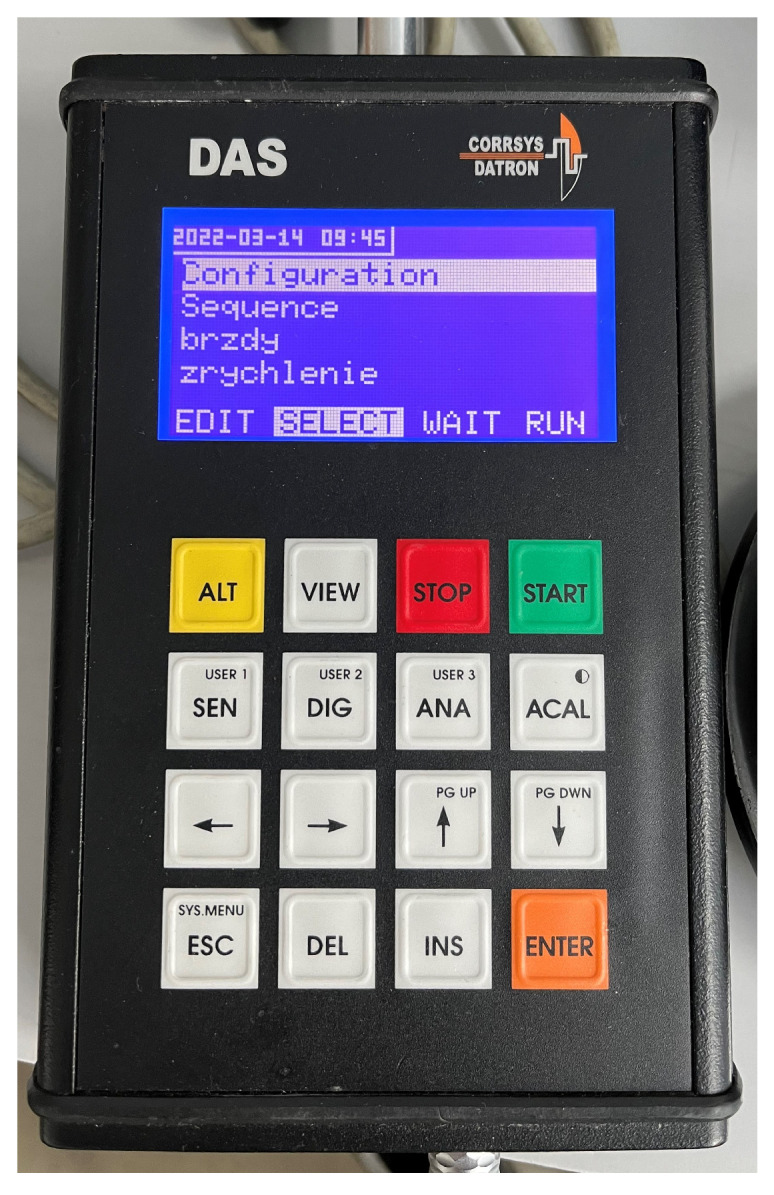
Control display.

The control display (Figure 13) serves as a control element for the entire device. It can be used to control the various measurement conditions and the initiation and termination of measurements. A small display is located in its upper part, where all the parameters and quantities being examined are displayed together with the device setting options. It can also be used to check the number of measurements that have already been taken. It can record and process input values in the range of –10 to 10 volts.

## 6. Results

As mentioned in the previous section, several series of measurements were performed using the Corrsys Datron DAS-3 apparatus to analyze the behavior of the single-axle trailer when driving over a road obstacle, especially the acceleration in the direction of the *x*-, *y*-, and *z*-axes.

The acceleration values when driving over the road obstacle are shown as tables and graphs to more easier visualized the results.

Two TAA-3206M4 acceleration sensors were used in the measurements, which were placed on the sides of the single-axle trailer directly above the wheels. Only the data measured by the sensor on the left side were used. The right sensor was incorrectly calibrated for the tests and therefore, it was not possible to obtain any data from this sensor.

### 6.1. Measurements Using 25 mm High Obstacle and Speed of 30 km/h

**Figure 14:**
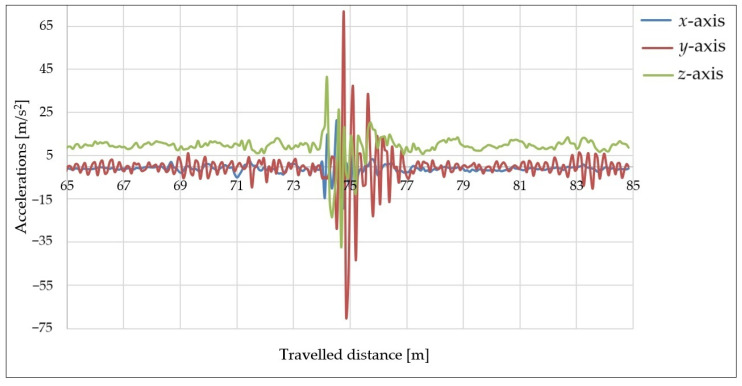
Acceleration curves at speed of 30 km/h with 300 kg load.

The first series of measurements was performed with a trailer load of 300 kg at four different speeds. The acceleration curves in the *x*-, *y*- and *z*-axes vs. the distance traveled at a speed of 30 km/h and with a tire pressure of 2.8 bar are shown in Figure 14.

Figure 14 shows the acceleration values of all phases of the travel of the trailer, i.e., driving on a flat road, approaching an obstacle, overcoming over the obstacle (loss of contact with the road surface), and the subsequent stabilization of the trailer. In this case, the sensor recorded the most significant acceleration values in the *y*-axis direction (orange color), i.e., in the direction perpendicular to the direction of vehicle movement, which was caused by the unsynchronized passage of the trailer’s wheels over the obstacle. After the trailer hit the road, the chassis components caused a springback. However, since the trailer was loaded, the course of the acceleration curve in the *z*-axis direction (green color) was not striking and the trailer stabilized within a short time.

**Figure 15:**
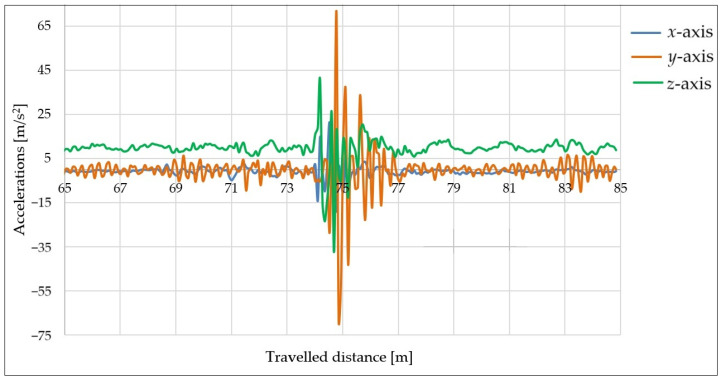
Acceleration curves at speed of 30 km/h with 0 kg load.

To compare the differences in the acceleration curves in the direction of all three axes, measurements were performed at the same speed with a different load (0 kg). The acceleration curves in the direction of the *x*-, *y*-, and *z*-axes at a speed of 30 km/h and with a tire pressure of 2.8 bar are depicted in Figure 15.

In this case, the most significant acceleration values were recorded in the *y*- and *z*-directions due to the trailer being empty. Its stabilization after hitting an obstacle and subsequent flight therefore took longer compared with the previous measurements, which can be seen in the acceleration curve in the *z*-axis direction (green color). The results shown in Figure 14 and Figure 15 showed similar values. This was attributed to the fact that the maximal payload of the trailer is 980 kg, i.e., more than three times the applied load. Therefore, the differences between the acceleration values are not very significant in these graphs.

#### 6.1.1. Measurements Using 25 mm High Obstacle, Speed of 30 km/h, and Underinflated Tires

The measurements were performed again using a shorter obstacle (height of 25 mm), a speed of 30 km/h, and a 300 kg load. The tire pressure was decreased from 2.8 to 1.6 bar.

**Figure 16:**
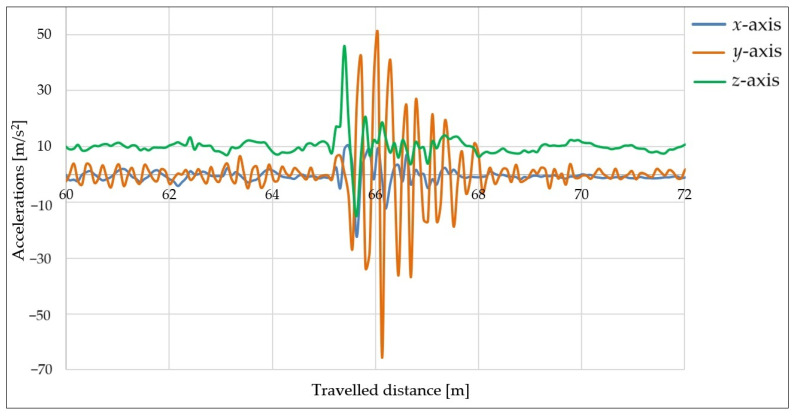
Acceleration curves with obstacle height of 25 mm, speed of 30 km/h, load of 300 kg, and underinflated tires.

The aim of this measurement series was to determine how underinflation of the trailer’s tires affects its behavior when hitting an obstacle. The acceleration curves in the direction of all axes vs. the distance traveled can be seen in Figure 16.

The most significant acceleration values (Figure 16) were recorded in the direction of the *y*- and *z*-axes (orange and green curves). The acceleration curve in the direction of the *x*-axis (blue color) was relatively flat. This occurred due to the underinflation of the tires. Since the tires were softer, the tires molded around the obstacle. The trailer stabilized within in a relatively small time window. The maximal acceleration values with underinflated tires were smaller than those with tires inflated to the prescribed pressure (the results in the previous section). This can be explained by the fact that lower air pressure in the tires increased their damping ability.

### 6.2. Measurements Using 25 mm High Obstacle, Speed of 30 km/h, and Different Loads

The next series of measurements was carried out using the same speed and tire pressure as the previous one, i.e., 30 km/h and 2.8 bar. The measurements were performed with 300, 150, and 0 kg loads.

**Figure 17:**
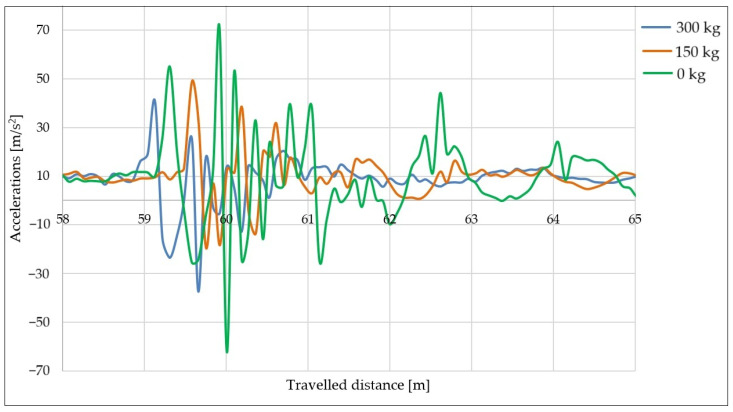
Acceleration curves in *z*-axis direction at speed of 30 km/h with different loads.

The aim of this series of measurements was to determine how the trailer load affects its behavior after hitting an obstacle. The acceleration curves in the *z*-axis direction vs. the distance traveled are shown in Figure 17.

The highest acceleration values (Figure 17) in the *z*-axis direction were recorded with an empty trailer (load of 0 kg). The empty trailer (green curve) appeared to be quite stiff and therefore, due to the effect of springback, it took the longest time to stabilize after hitting the obstacle. With the 150 and 300 kg loads, the impact to the trailer appeared to be significantly softer, with the curves (orange and blue) showing lower acceleration values. The time required for the trailer to stabilize after hitting the obstacle was also significantly reduced with a load. The difference in the measured acceleration values for the trailer with a 300 kg load and the empty trailer (0 kg) showed an almost twofold difference.

#### 6.2.1. Measurement of Acceleration in x-Axis Direction When Passing over 25 mm High Obstacle at Speed of 30 km/h with 300 kg Load 

In this series of measurements, the speed and load of the trailer were the same as in the previous case (30 km/h and 300 kg). The data from both TAA-3206M4 sensors mounted on the sides of the trailer (L—left side; R—right side) and from the K-BEAM 8304A10 sensor mounted on the towing device were used.

**Figure 18:**
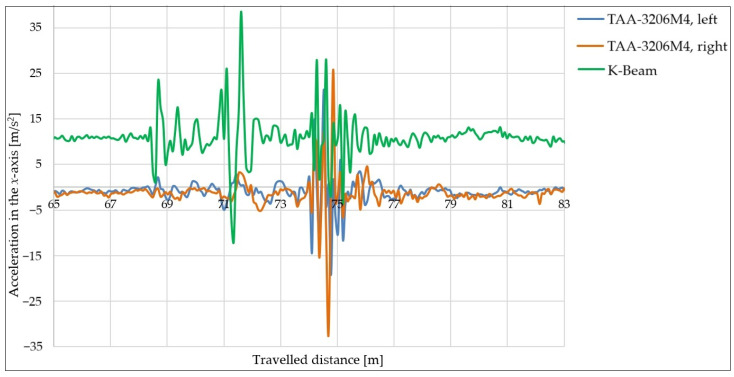
Acceleration curves in *x*-axis direction with obstacle height of 25 mm, speed of 30 km/h, and 300 kg load from three different sensors on the trailer.

The individual acceleration curves in the *x*-axis direction vs. the travelled distance can be seen in Figure 18.

Figure 18 shows how the trailer pushes on the towing vehicle as it moves. The initial fluctuations in the green curve (the K-BEAM sensor), visible between 67 and 73 m of the track, were caused by driving downhill, braking, and the subsequent acceleration. Fluctuations in the measured acceleration values in the *x*-axis direction were also observed in the area of impact with the obstacle.

When the trailer was loaded, a significant force acted on the towing vehicle at the moment of impact and subsequent rebound, which gradually decreased as the trailer returned to its starting position.

### 6.3. Measurements Using 50 mm High Obstacle and Speed of 20 km/h

Next, measurements were performed using a taller obstacle with a height of 50 mm. Due to concerns about possible damage to the axles of the vehicle combination, the speed was reduced to 20 km/h. The tire pressure remained unchanged at 2.8 bar and the load used was 200 kg.

**Figure 19:**
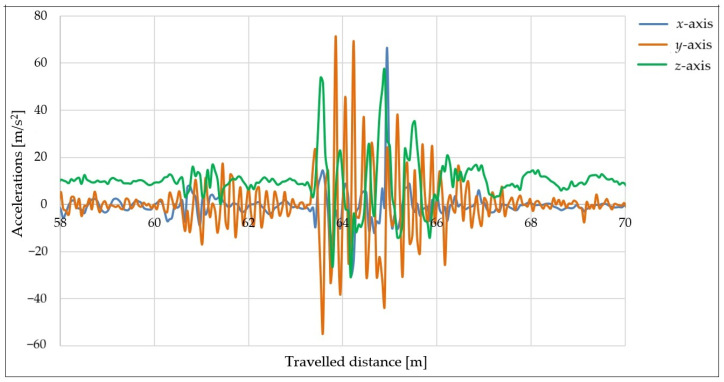
Acceleration curves measured in *x*-, *y*-, and *z*-axis directions using 50 mm high obstacle, speed of 20 km/h, and different loads.

The aim of these measurements was to determine how the height of the obstacle affects the behavior of the trailer after hitting the obstacle. The acceleration curves in the directions of the three axes vs. the distance traveled can be seen in Figure 19.

Figure 19 shows that the impact with the taller obstacle caused a significant oscillation in the trailer in the *y*-axis (orange curve). After the impact, large fluctuations occurred in the *x-* and *z*-axis directions (blue and green curves), which were caused by the trolley bouncing off the road again since the wheels did not hit the road at the same time. The stabilization of the trailer after the impact required a longer time window compared with driving over a shorter obstacle.

## 7. Discussion

The aim of this research was to assess the influence of various factors (speed, trailer load, tire pressure of the trailer, and height of the obstacle) on the behavior of a trailer during and after driving over a simulated road obstacle. The obtained data showed the change in acceleration and the distance required to stabilize the trailer after hitting the obstacle. These results could be used to identify the conditions for the safe use of trailers.

When using the shorter obstacle (25 mm) and an empty trailer, the acceleration curves sharply increased, especially along the *y*- and *z*-axes. The acceleration value depended on the speed of the trailer. Since the trailer was empty, its movements appeared to be quite stiff, so at higher speeds, after hitting the obstacle, the wheels lost contact with the road surface (the trailer flew), followed by an impact and rebound, after which, it gradually stabilized. This phenomenon was caused by the suspension of the trailer after the impact. In the case of a loaded trailer, a smaller increase in the acceleration values along the *z*-axis were observed due to the load, which acted on the suspension of the trailer. The time window and distance after the impact required for the trailer to stabilize were also significantly shorter in comparison with the empty trailer.

When using the taller obstacle (50 mm) and a loaded trailer, there was a sharp increase in the acceleration values in the *x*-axis direction. This was caused by the sharp impact of the wheel supporting a larger mass as it hit a taller obstacle. In this case, the force acting on the wheels of the trailer when hitting the obstacle also increased compared with that when hitting the shorter obstacle.

When the tire pressure was reduced to 1.6 bar, a remarkable phenomenon was observed. The changes in the measured acceleration values in the *x*-axis direction were relatively small due to the underinflation (softening) of the wheels, which molded around the obstacle. Due to the underinflated tires, there was also a significant decrease in the force acting on the wheels when hitting the obstacle.

Future research in this field should focus on creating a multibody model of a vehicle–trailer combination. The research team has experience with this type of model for rail vehicles [55]. The results of simulations can be compared with the results from experiments similar to those performed in the present study. The simulation models can be used for dynamics and strength analyses [56,57,58,59]. This approach is widely applied not only to vehicles, but also in other technical fields [60]. Once a multibody model is established and verified, it can be used to perform simulations and analyses of vehicle–trailer combinations driving over obstacles at high driving speeds that would be dangerous to perform with a real vehicle [61]. Furthermore, it will be also possible to change parameters and the shape of the obstacle to reflect obstacles that occur in the real world. Such simulations will be more time-efficient and safer.

The position of the center of gravity (CoG) of the load in a trailer is another important parameter that affects the driving properties of a vehicle–trailer combination [62,63,64]. The improper loading of a trailer can lead to a serious accident. This applies not only to vehicle–trailer combinations [28], but also to articulated buses, as demonstrated in [65,66,67,68]. The proposed simulations in this field will also allow researchers to perform studies on this parameter. This is where the advantages of simulations becomes apparent as it removes the risk of material damage during investigations.

Another possible future direction for research is the design of a simulator that would allow researchers to perform experimental tests. This simulator would include a vehicle and a trailer, where the user could set the load, tire pressure [69], height of the obstacle, and driving speed. Moreover, the simulator could also allow the position of the load on a trailer and the towing direction (longitudinal and lateral directions) to be changed to investigate the influence of the center of gravity position on the driving properties. It would also be useful to be able to change the wheel track and wheelbase along with changing the drawbar length to simulate the wide range of vehicles and trailers that exist.

## 8. Conclusions

Trailers towed by a passenger car are often used to transport materials or as a caravan for short- and long-distance trips. Trailers cause additional loads on the towing vehicle. When these vehicles drive over road irregularities or road obstacles, the load on the towing vehicle is even more significant. Therefore, the factors that could have negative effects on the vehicles need to be assessed. The present research performed experimental measurements of a vehicle–trailer combination as it drove over a road obstacle. The vehicle–trailer combination consisted of a passenger motor vehicle as the towing vehicle and a freight flatbed trailer. The acceleration of the trailer structure was measured during the test. Several different loads and driving speeds and two road obstacle heights were used. Based on the results, it can be concluded that these factors affected the behavior of the trailer when driving over an uneven surface, i.e., its driving characteristics.

## Figures and Tables

**Table 1:** Parameters for test series No. 1. Results are shown in Section 6.1.

Parameter	Unit	Value
Driving speed	km/h	30
Load	kg	0, 300
Tire pressure	bar	2.8
Obstacle height	mm	25

**Table 2:** Parameters for test series No. 2. Results are shown in Section 6.1.1.

Parameter	Unit	Value
Driving speed	km/h	30
Load	kg	0, 300
Tire pressure	bar	1.6
Obstacle height	mm	25

**Table 3:** Parameters for test series No. 3. Results are shown in Section 6.2 and Section 6.2.1.

Parameter	Unit	Value
Driving speed	km/h	30
Load	kg	0, 150, 300
Tire pressure	bar	1.6
Obstacle height	mm	25

**Table 4:** Parameters for test series No. 4. Results are shown in Section 6.3.

Parameter	Unit	Value
Driving speed	km/h	20
Load	kg	0, 200
Tire pressure	bar	2.8
Obstacle height	mm	50

**Table 5:** Technical parameter of towing vehicle.

Engine	1.6 VVT
Fuel	Gasoline
Max. power/rpm [kW/min^−1^]	88/6200
Max. torque/rpm [N∙m/min^−1^]	156/4400
Drive	4WD
**Dimension**	**[mm]**
Total length	4175
Total width	1775
Toal height	1610
Wheelbase	2500
Front wheel track	1635
Rear wheel track	1505
Tires	215/55 R17
**Mass**	**[kg]**
Curb weight	1275
Total weight	1730

**Table 6:** Technical parameters of single-axle trailer.

Type	Braked Flatbed Trailer
Number of axles	1
**Dimension**	**[mm]**
Total length	3600
Total width	1750
Toal height	935
Wheel track	1550
Tires	185/65 R14
**Mass**	**[kg]**
Curb weight	277
Total weight	1300B

## Data Availability

The data are contained within the article.
